# Supplementary material for: The Impact of Emerging Plasmodium knowlesi on Accurate Diagnosis by Light Microscopy: A Systematic Review and Modeling Analysis
Source: Am J Trop Med Hyg. 2022 Dec 12;108(1):61–8. doi: 10.4269/ajtmh.21-1155 (PMC9833074; doi:10.4269/ajtmh.21-1155)
Supplement: Supplementary file 1 [file tpmd211155.SD1.pdf]

SUPPORTING INFORMATION

The impact of emerging *Plasmodium knowlesi* on accurate diagnosis by light microscopy: a systematic review and modelling analysis

John H. Huber, Margaret Elliot, Cristian Koepfli, T. Alex Perkins

Department of Biological Sciences and Eck Institute for Global Health, University of Notre Dame, Notre Dame, IN, USA

Corresponding Author: huber.j.h@wustl.edu (JHH)

Supplemental Methods

Systematic Review

Our search of MEDLINE and Web of Science returned 275 studies published before April 30th, 2021. After removing duplicates, we screened the abstracts of 176 studies. Of these, 101 full-text articles were then assessed for eligibility. Of the 75 studies that were excluded upon abstract screening, 50 were not primary research article and were instead case reports (n = 28), reviews (n = 14), conference abstracts (n = 3), editorials (n = 2), book chapters (n = 1), clinical guidelines n = 1), or study protocols (n = 1). The remaining studies excluded upon abstract screening did not compare diagnostic methods (n = 13), did not include *P. knowlesi* (n = 5), were conducted in a non-endemic location (n = 3), did not include clinical samples (n = 2), did not use a PCR protocol that targeted all five *Plasmodium* spp. (n = 1), or was a supplementary table to previously identified study (n = 1).

Of the 101 full-text articles that were assessed for eligibility, 10 met the inclusion criteria. The remaining 89 full-text articles were excluded for the following reasons: no comparison of diagnostic methods (n = 20); study conducted in non-endemic region (n = 12); no two-way table provided (n = 11); no presence of *P. knowlesi* (n = 10); review article (n = 8); no clinical samples (n = 7); case report (n = 4); conference abstract (n = 4); full-text unavailable (n = 4); PCR protocol could not target all five *Plasmodium* spp. (n = 3); *Plasmodium* spp. undefined (n = 2); overlapping data set with other identified publication (n = 2); no co-circulation of *Plasmodium* spp. parasites (n = 1); study not in English (n = 1); study conducted prior to *P. knowlesi* being recognized as public health threat (n = 2);

Prisma Checklist

Section and Topic	Item #	Checklist item	Location where item is reported
**TITLE**			
Title	1	Identify the report as a systematic review.	1
**ABSTRACT**			
Abstract	2	See the PRISMA 2020 for Abstracts checklist.	2
**INTRODUCTION**			
Rationale	3	Describe the rationale for the review in the context of existing knowledge.	7
Objectives	4	Provide an explicit statement of the objective(s) or question(s) the review addresses.	7
**METHODS**			
Eligibility criteria	5	Specify the inclusion and exclusion criteria for the review and how studies were grouped for the syntheses.	8
Information sources	6	Specify all databases, registers, websites, organisations, reference lists and other sources searched or consulted to identify studies. Specify the date when each source was last searched or consulted.	7
Search strategy	7	Present the full search strategies for all databases, registers and websites, including any filters and limits used.	7
Selection process	8	Specify the methods used to decide whether a study met the inclusion criteria of the review, including how many reviewers screened each record and each report retrieved, whether they worked independently, and if applicable, details of automation tools used in the process.	7-8, S2
Data collection process	9	Specify the methods used to collect data from reports, including how many reviewers collected data from each report, whether they worked independently, any processes for obtaining or confirming data from study investigators, and if applicable, details of automation tools used in the process.	8
Data items	10a	List and define all outcomes for which data were sought. Specify whether all results that were compatible with each outcome domain in each study were sought (e.g. for all measures, time points, analyses), and if not, the methods used to decide which results to collect.	8-9
	10b	List and define all other variables for which data were sought (e.g. participant and intervention characteristics, funding sources). Describe any assumptions made about any missing or unclear information.	8-9
Study risk of bias assessment	11	Specify the methods used to assess risk of bias in the included studies, including details of the tool(s) used, how many reviewers assessed each study and whether they worked independently, and if applicable, details of automation tools used in the process.	10, S13-S14
Effect measures	12	Specify for each outcome the effect measure(s) (e.g. risk ratio, mean difference) used in the synthesis or presentation of results.	10
Synthesis methods	13a	Describe the processes used to decide which studies were eligible for each synthesis (e.g. tabulating the study intervention characteristics and comparing against the planned groups for each synthesis (item #5)).	7-8, S2
	13b	Describe any methods required to prepare the data for presentation or synthesis, such as handling of missing summary statistics, or data conversions.	NA
	13c	Describe any methods used to tabulate or visually display results of	NA

Section and Topic	Item #	Checklist item	Location where item is reported
		individual studies and syntheses.	
	13d	Describe any methods used to synthesize results and provide a rationale for the choice(s). If meta-analysis was performed, describe the model(s), method(s) to identify the presence and extent of statistical heterogeneity, and software package(s) used.	8-10
	13e	Describe any methods used to explore possible causes of heterogeneity among study results (e.g. subgroup analysis, meta-regression).	10, S13-S17
	13f	Describe any sensitivity analyses conducted to assess robustness of the synthesized results.	10, S13-S17
Reporting bias assessment	14	Describe any methods used to assess risk of bias due to missing results in a synthesis (arising from reporting biases).	S13-S17
Certainty assessment	15	Describe any methods used to assess certainty (or confidence) in the body of evidence for an outcome.	S7-S12
**RESULTS**			
Study selection	16a	Describe the results of the search and selection process, from the number of records identified in the search to the number of studies included in the review, ideally using a flow diagram.	10-11, S2
	16b	Cite studies that might appear to meet the inclusion criteria, but which were excluded, and explain why they were excluded.	S2
Study characteristics	17	Cite each included study and present its characteristics.	11, S9
Risk of bias in studies	18	Present assessments of risk of bias for each included study.	S13-S14
Results of individual studies	19	For all outcomes, present, for each study: (a) summary statistics for each group (where appropriate) and (b) an effect estimate and its precision (e.g. confidence/credible interval), ideally using structured tables or plots.	10-15, S9-S12
Results of syntheses	20a	For each synthesis, briefly summarise the characteristics and risk of bias among contributing studies.	S13-S14
	20b	Present results of all statistical syntheses conducted. If meta-analysis was done, present for each the summary estimate and its precision (e.g. confidence/credible interval) and measures of statistical heterogeneity. If comparing groups, describe the direction of the effect.	10-15, S9-S12
	20c	Present results of all investigations of possible causes of heterogeneity among study results.	16-17, S13-S17
	20d	Present results of all sensitivity analyses conducted to assess the robustness of the synthesized results.	S13-S17
Reporting biases	21	Present assessments of risk of bias due to missing results (arising from reporting biases) for each synthesis assessed.	NA
Certainty of evidence	22	Present assessments of certainty (or confidence) in the body of evidence for each outcome assessed.	S7-S12
**DISCUSSION**			
Discussion	23a	Provide a general interpretation of the results in the context of other evidence.	17-19
	23b	Discuss any limitations of the evidence included in the review.	19
	23c	Discuss any limitations of the review processes used.	19
	23d	Discuss implications of the results for practice, policy, and future	18

Section and Topic	Item #	Checklist item	Location where item is reported
		research.	
**OTHER INFORMATION**			
Registration and protocol	24a	Provide registration information for the review, including register name and registration number, or state that the review was not registered.	NA
	24b	Indicate where the review protocol can be accessed, or state that a protocol was not prepared.	NA
	24c	Describe and explain any amendments to information provided at registration or in the protocol.	NA
Support	25	Describe sources of financial or non-financial support for the review, and the role of the funders or sponsors in the review.	10
Competing interests	26	Declare any competing interests of review authors.	10
Availability of data, code and other materials	27	Report which of the following are publicly available and where they can be found: template data collection forms; data extracted from included studies; data used for all analyses; analytic code; any other materials used in the review.	21

Accounting for Differences in Study Design

Some studies in our systematic review reported samples as an "*P. knowlesi* / *P. malariae*" LM diagnosis, indicating that the microscopist identified *P. knowlesi* and/or *P. malariae* parasites in the sample but could not make a more precise mono-infection or co-infection diagnosis. We accounted for this in the latent class model by computing the union of all possible outcomes that could lead to a "*P. knowlesi* / *P. malariae*", given a certain level of sensitivity and specificity of PCR diagnosis. This accounts for true and false positives for both *P. knowlesi* and *P. malariae* parasites.

In our analysis, we considered two modeling scenarios. In the first, we assumed that no study could accurately distinguish between *P. knowlesi* and *P. malariae* by light microscopy. We therefore grouped all LM samples for *P. knowlesi* and *P. malariae* and estimate the sensitivities of LM for *"P. knowlesi / P. malariae"* given that the sample was truly either *P. knowlesi* or *P. malariae* by PCR using the described methods above. In our second scenario, we assumed that analyses that reported *P. knowlesi* and *P. malariae* by LM separately could distinguish them with

specific sensitivities and specificities. We thus aimed to estimate these sensitivities and specificities of LM for *P. knowlesi* and *P. malariae.*

Hierarchical Model

We fit our hierarchical latent class model to the studies identified in our systematic review to estimate the study-level sensitivities and specificities of LM as well as the hierarchical distributions for the five *Plasmodium* spp. Using eq. (1) in the main text, we computed the probability of observing a set of LM outcomes $\vec{o}^{(l)}{}_{LM} = \left\{o_{LM}^{(k,l)}\right\}$ and PCR outcomes $\vec{o}^{(l)}{}_{PCR} = \left\{o_{PCR}^{(k,l)}\right\}$ in study *l* across all *Plasmodium* spp. as

$$\Pr\left(\vec{o}^{(l)}{}_{LM}, \vec{o}^{(l)}{}_{PCR} \middle| \overrightarrow{se}^{(l)}{}_{LM}, \overrightarrow{sp}^{(l)}{}_{LM}, \vec{\theta}^{(l)}\right) = \prod_{k \in \{Pf,Pv,Pk,Pm,Po\}} p_{o_{LM}^{(k,l)} o_{PCR}^{(k,l)} k}. \quad (S1)$$

In eq. (S1), $\overrightarrow{se}^{(l)}{}_{LM}$ and $\overrightarrow{sp}^{(l)}{}_{LM}$ are the vectors of LM sensitivities and specificities for all *Plasmodium* spp. in study *l*, and $\vec{\theta}^{(l)}$ is the vector of prevalence for the *Plasmodium* spp. in study *l*. The individual probabilities in the product of eq. (S1) are computed using eq. (1).

Using eq. (1) and eq. (S1) and accounting for features of the study design, we computed a probability vector $\vec{p}^{(l)}$ for each study *l*, where each element corresponds to the probability of observing a set of LM and PCR diagnostic outcomes across all *Plasmodium* spp. in that study. Given a vector $\vec{s}^{(l)}$ containing the number of samples observed in study *l* for each corresponding set of LM and PCR diagnostic outcomes, we defined the likelihood of the model given the data as

$$\mathcal{L}\left(\overrightarrow{se}_{LM}, \overrightarrow{sp}_{LM} \middle| \{\vec{s}^{(1)}, \dots, \vec{s}^{(N)}\}, \{\vec{\theta}^{(1)}, \dots, \vec{\theta}^{(N)}\}\right) = \prod_{l=1}^{N} \text{Multinomial}\left(\vec{s}^{(l)} \middle| \vec{p}^{(l)}\right). \quad (S2)$$

We further defined parameters $\mu_{se}^{(k)}$ and $\sigma_{se}^{(k)}$ and $\mu_{sp}^{(k)}$ and $\sigma_{sp}^{(k)}$, which comprised the hierarchical distributions for sensitivity and specificity for each *Plasmodium* spp. *k*. These distributions were defined on the logit scale, such that the study-level effects were computed as $Normal\left(logit\left(se_{LM}^{(k,l)}\right)\middle|\mu_{se}^{(k)}, \sigma_{se}^{(k)}\right)$ and $Normal\left(logit\left(sp_{LM}^{(k,l)}\right)\middle|\mu_{sp}^{(k)}, \sigma_{sp}^{(k)}\right)$ for each *Plasmodium* spp. *k* and study *l*. In the modeling scenario in which we grouped *P. knowlesi* and *P. malariae* LM diagnoses, the sampler could not stably estimate the study-level effects of *P. knowlesi* and *P. malariae* due to identifiability issues. As a result, for this model scenario, we only report study-level effects for *P. falciparum*, *P. vivax*, and *P. ovale*.

We used the No-U-Turn sampler provided in Stan to fit the model. The No-U-Turn sampler is an adaptive version of the Hamiltonian Monte Carlo algorithm that is capable of sampling from a high-dimensional posterior distribution. We assumed uniform prior distributions on [0,1] for the study-level sensitivities and specificities, uniform prior distributions on (-∞,∞) for the hierarchical means, and half-normal prior distributions with zero mean and standard deviation of 0.25 on the hierarchical standard deviations. We evaluated the sensitivity of our hierarchical parameter estimates to the assumed standard deviation of this half-normal distribution.

Simulation Study

To confirm that our inference framework could estimate each parameter with appropriate uncertainty, we performed a simulation study. We simulated 200 synthetic data sets, each

consisting of 10 studies that matched the study designs of the 10 studies identified in our systematic review. For each synthetic data set, we sampled parameter sets from the respective prior distributions and simulated the data by using the likelihood of our model as a data-generating process. We then ran 4 chains with 2,000 samples each using the No-U-Turn Sampler in Stan with a warm-up period of 1,000 samples to obtain a posterior distribution of 4,000 samples for each synthetic data set. We assessed the performance of our inference framework by visually comparing the true values for each parameter to the posterior estimates that we obtained and by computing the coverage probabilities (i.e., the proportion of synthetic data sets for which the true value of each parameter is within the posterior credible interval). When calculated using the 95% credible interval, the coverage probability should be close to 0.95.

The results of our simulation study revealed that our inference framework could generally accurately estimate the parameters of our model (Figs. S1–S2). For the modeling scenario in which we grouped *P. knowlesi* and *P. malariae* by LM, we accurately estimated the means of the hierarchical distributions for sensitivity (Fig. S1A) and specificity (Fig. S1C), and the ranges of the coverage probabilities across the *Plasmodium* spp. was 0.91 – 0.95 and 0.95 – 0.97, respectively. There was greater uncertainty on our posterior estimates of hierarchical standard deviation for sensitivity (Fig. S1B) and specificity (Fig. S1D). Nevertheless, the range of the coverage probabilities across the *Plasmodium* spp. was 0.93 – 0.95 for the hierarchical standard deviation for sensitivity and 0.95 – 0.98 for the hierarchical standard deviation for specificity. In general, for study-level sensitivities and specificities, we observed good agreement between our posterior estimates that we obtained and the true values. There was greater uncertainty for our estimates for *P. knowlesi, P. malariae, and P. ovale* than our estimates for *P. falciparum* and *P. vivax* due to the lower observed PCR prevalence for these species. Nevertheless, the median (and

range) coverage probabilities across the studies were 0.95 (0.93 – 0.98) for *P. falciparum*, 0.95 (0.93 – 0.96) for *P. vivax*, 0.96 (0.92 – 0.98) for *P. knowlesi*, 0.95 (0.90 – 0.98) for *P. malariae*, and 0.92 (0.89 – 0.93) for *P. ovale*. For study-level specificities, we also observed generally good agreement between our posterior estimates and the true values. The coverage probabilities were 0.95 (0.89 – 0.98) for *P. falciparum*, 0.95 (0.93 – 0.96) for *P. vivax*, 0.95 (0.93 – 0.98) for *P. knowlesi*, 0.96 (0.94 – 0.97), and 0.96 (0.93 – 0.99) for *P. ovale*.

We obtained comparable accuracies in our simulation sweep under our second modeling scenario in which we separated *P. knowlesi* and *P. malariae* LM samples where available (Fig. S2)

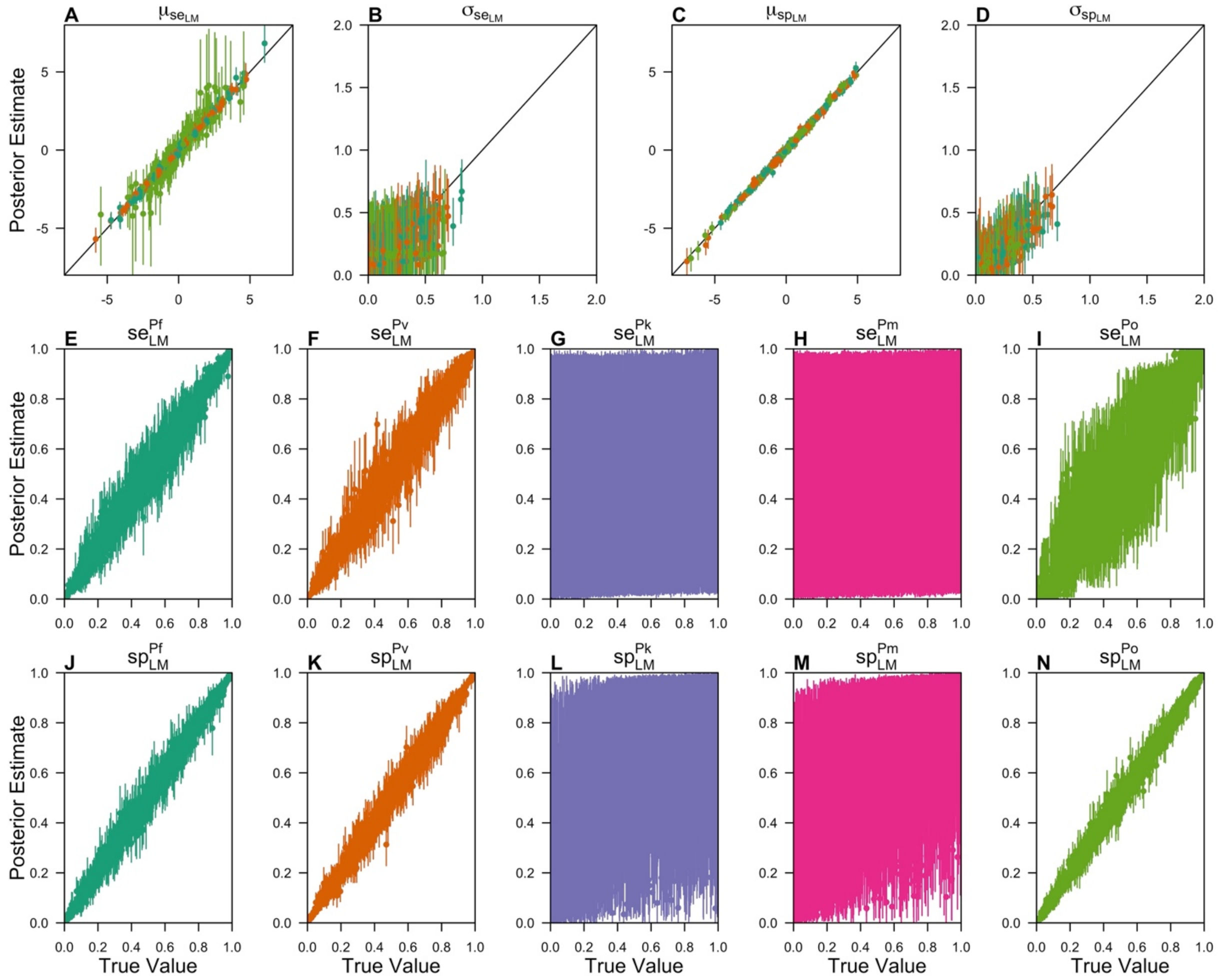

Figure S1. Scatterplot comparison of true and inferred values from simulation study in which P. knowlesi and P. malariae LM samples are grouped. *Comparisons of true and inferred posterior values are shown for the hierarchical (A) mean and (B) standard deviation of LM sensitivity, hierarchical (C) mean and (D) standard deviation of LM specificity, (E-I) study-level LM sensitivities, and (J-N) study-level LM specificities. In each plot, the point is the median posterior estimate, and the segment is the 95% credible interval. The color signifies the Plasmodium spp., with teal representing P. falciparum, orange representing P. vivax, purple representing P. knowlesi, pink representing P. malariae, and green representing P. ovale.*

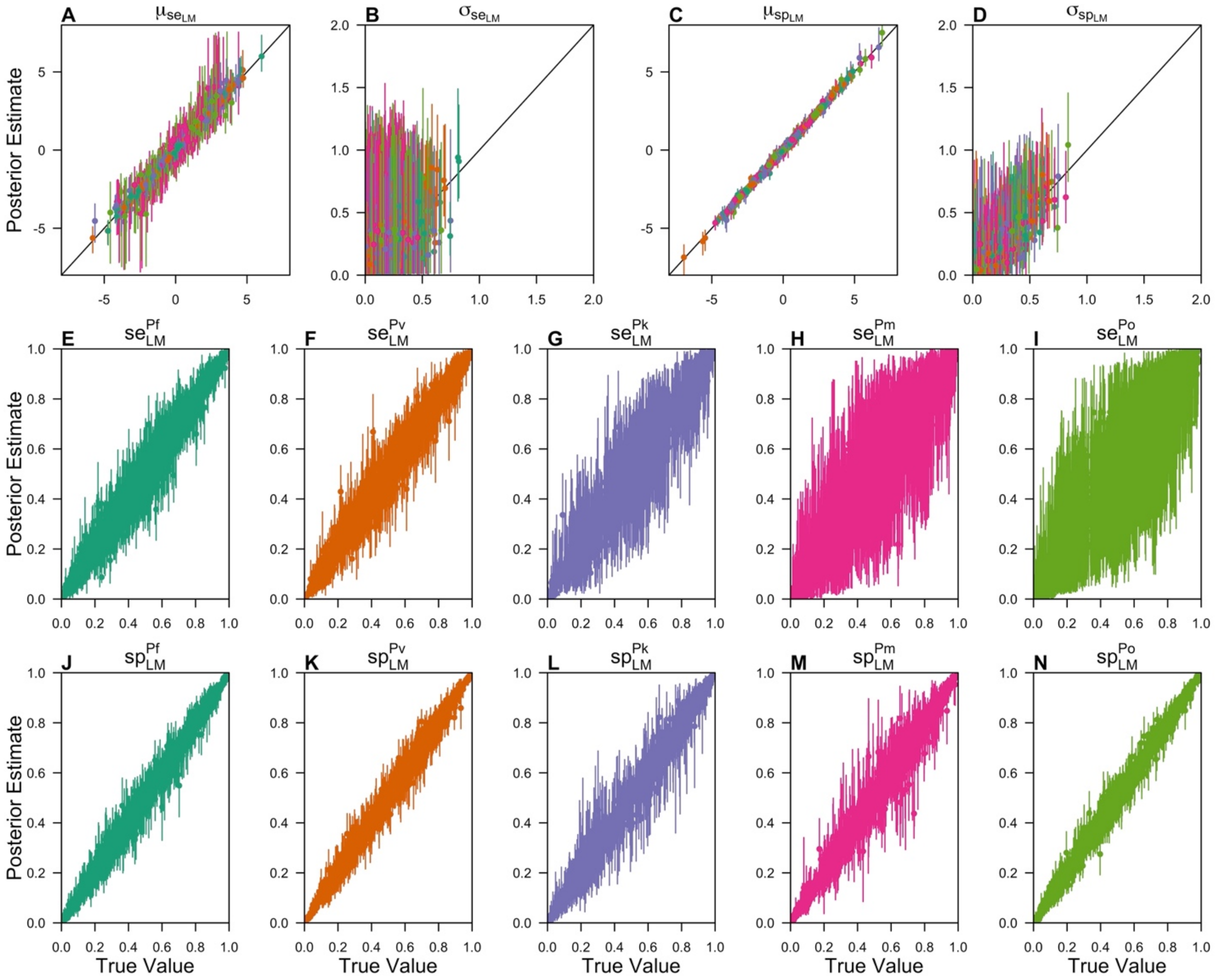

Figure S2. Scatterplot comparison of true and inferred values from simulation study in which P. knowlesi and P. malariae LM samples are not grouped. *Comparisons of true and inferred posterior values are shown for the hierarchical (A) mean and (B) standard deviation of LM sensitivity, hierarchical (C) mean and (D) standard deviation of LM specificity, (E-I) study-level LM sensitivities, and (J-N) study-level LM specificities. In each plot, the point is the median posterior estimate, and the segment is the 95% credible interval. The color signifies the Plasmodium spp., with teal representing P. falciparum, orange representing P. vivax, purple representing P. knowlesi, pink representing P. malariae, and green representing P. ovale.*

Results in which *P. knowlesi* and *P. malariae* LM Samples are Not Grouped

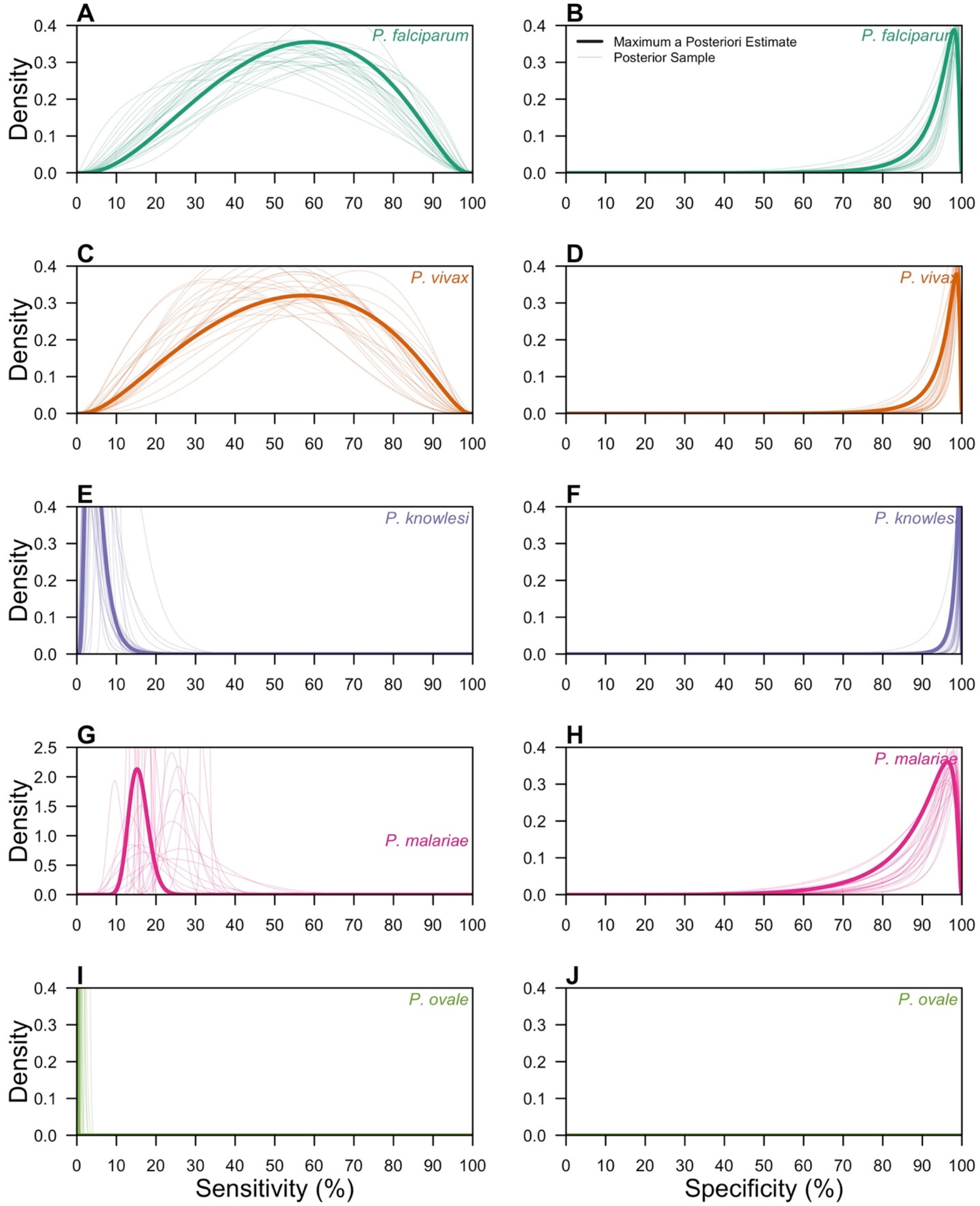

Figure S3. Group-level posterior estimates of LM diagnostic performance in which P. knowlesi and P. malariae LM samples are not grouped. *The group-level distributions of LM sensitivity (A, C, E, G, I) and LM specificity (B, D, F, H, J) are shown for Plasmodium falciparum (teal; A, B), Plasmodium vivax (orange; C, D), Plasmodium knowlesi (purple; E, F),*

Plasmodium malariae (pink; G, H), and Plasmodium ovale (green; I, J). Thick lines are the maximum a posteriori estimates, and thin lines are 25 posterior samples.

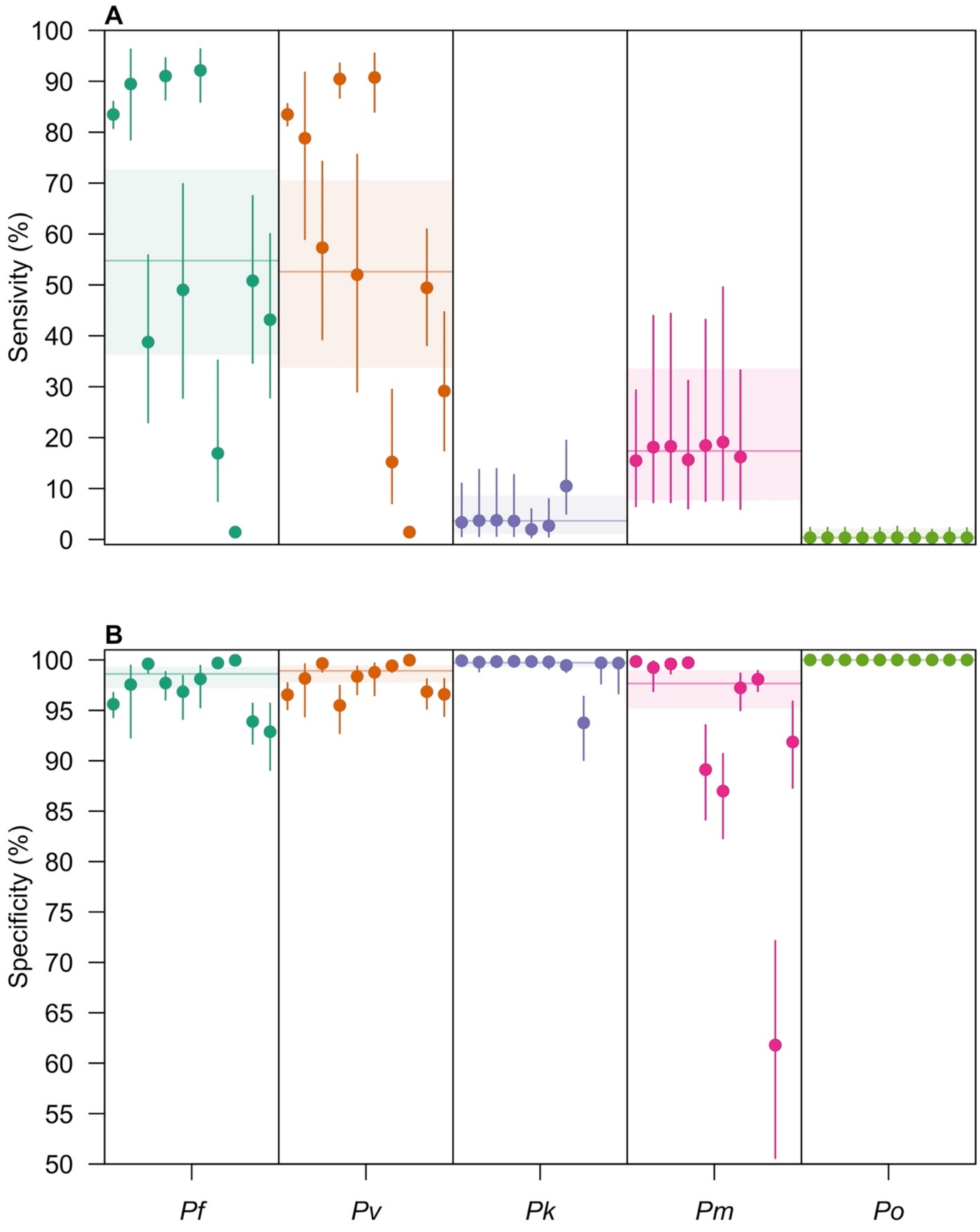

Figure S4. Site-level posterior estimates of LM diagnostic performance in which P. knowlesi and P. malariae LM samples are not grouped. *The site-level posterior estimates of (A) LM sensitivity and (B) LM specificity are shown for P. falciparum (teal), P. vivax (orange), P. knowlesi (purple), P. malariae (pink), and P. ovale (green). Circles are the median posterior estimate, and the vertical segment is the 95% credible interval. The horizontal line is the posterior median of the group-level mean, and the horizontal shaded region is the corresponding 95% credible interval.*

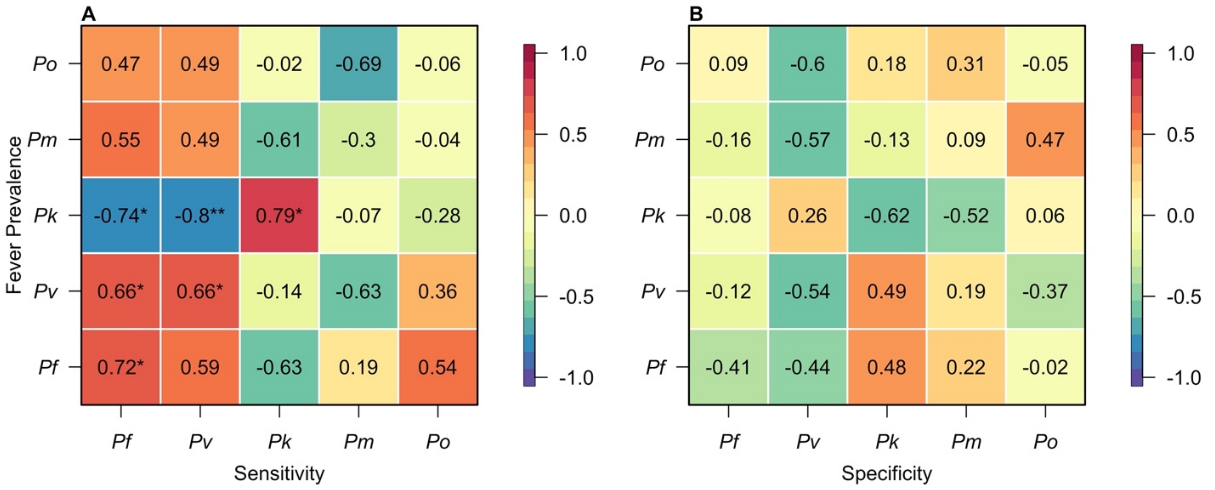

Figure S5. Correlations between Plasmodium spp. fever prevalence and LM diagnostic performance. *The correlations between Plasmodium spp. fever prevalence and (A) LM sensitivity and (B) LM specificity are reported. The color represents the strength of the correlation, with red colors denoting positive correlations and blue colors denoting negative correlations. '*' denotes p < 0.05, '**' denotes p < 0.01, and '***' denotes p < 0.001.*

Systematic Review

Table S1. Characteristics of Included Studies.

Author	Publication Year	Location	Collection	Enrollment Criteria	Pk/Pm	Samples
**Cooper *et al.***	2020	Malaysia	Jan. 2015 – Dec. 2017	PCR- and LM-Positive	Yes	3,541
**Han *et al.***	2017	Myanmar	2013 – 2015	None	No	90
**Baum *et al.***	2016	Thailand	Mar. 2012 – Jul. 2012	None	No	297
**Chua *et al.***	2015	Malaysia	2008 – 2010	None	No	227
**Yusof *et al.***	2014	Malaysia	Sep. 2012 – Dec. 2013	LM-Positive	No	457
**Zhou *et al.***	2014	China	Jan. 2008 – Aug. 2012	None	No	560
**Barber *et al.***	2013	Malaysia	Sep. 2010 – Oct. 2011	PCR- and LM-Positive	Yes	303
**Goh *et al.***	2013	Malaysia	2008 – 2011	LM-Positive	No	189
**Barber *et al.***	2012	Malaysia	Jan. 2009 – Nov. 2011	LM-Positive	Yes	461
**Putaporntip *et al.***	2009	Thailand	Oct. 2006 – Sep. 2007	None	No	1,874

Data Analysis

Summary of Results

Table S2. Posterior estimates and convergence statistics of group-level effects when *P. knowlesi* and *P. malariae* LM samples are grouped.

Parameter	Posterior Estimate (95% CI)	Effective Sample Size	R-Hat
$\boldsymbol{\mu_{se}}$			
P. falciparum	0.651 (-0.077 – 1.406)	942.8	1.001
P. vivax	0.518 (-0.162 – 1.218)	865.4	1.008
P. ovale	-5.244 (-8.467 - -3.439)	2729.7	1.000
$\boldsymbol{\sigma_{se}}$			
P. falciparum	1.095 (0.886 – 1.357)	2125.7	1.001
P. vivax	1.068 (0.859 – 1.327)	1722.4	1.000
P. ovale	0.174 (0.007 – 0.575)	4346.5	1.000
$\boldsymbol{\mu_{sp}}$			
P. falciparum	4.007 (3.333 – 4.676)	873.0	1.002
P. vivax	4.273 (3.569 – 4.954)	991.8	1.002
P. ovale	11.945 (10.114 – 15.422)	2966.6	1.000
$\boldsymbol{\sigma_{sp}}$			
P. falciparum	0.967 (0.753 – 1.229)	1972.3	1.002
P. vivax	0.967 (0.766 – 1.236)	2358.5	1.001
P. ovale	0.177 (0.007 – 0.607)	3738.2	1.001

Table S3. Posterior estimates and convergence statistics of study-level sensitivities when *P. knowlesi* and *P. malariae* LM samples are grouped.

Parameter	Posterior Estimate (95% CI)	Effective Sample Size	R-Hat
$se_{LM}^{(Pf)}$			
Putaporntip *et al.*	83.5% (80.7 – 86.0%)	4211	1.001
Han *et al.*	89.8% (78.5 – 96.4%)	5511	1.000
Baum *et al.*	40.2% (23.6 – 58.3%)	7895	1.000
Cooper *et al.*	2.21% (1.32 – 3.55%)	2372	1.002
Zhou *et al.*	91.1% (86.1 – 94.8%)	6566	1.000
Barber *et al.*	52.4% (36.5 – 68.6%)	8555	1.000
Barber *et al.*	48.2% (32.0 – 65.6%)	2878	1.001
Goh *et al.*	65.9% (48.1 – 81.4%)	4334	0.999
Chua *et al.*	92.4% (86.1 – 96.4%)	7362	1.000
Yusof *et al.*	79.3% (62.7 – 91.4%)	5016	1.000
$se_{LM}^{(Pv)}$			
Putaporntip *et al.*	83.5% (81.3 – 85.5%)	3979	1.000
Han *et al.*	79.7% (59.5 – 92.4%)	5587	0.999
Baum *et al.*	58.4% (39.8 – 75.4%)	7554	1.000
Cooper *et al.*	2.20% (1.32 – 3.60%)	2572	1.000
Zhou *et al.*	90.5% (86.8 – 93.4%)	5494	1.000
Barber *et al.*	50.5% (39.5 – 62.1%)	8018	0.999
Barber *et al.*	33.0% (20.0 – 48.6%)	4049	1.000
Goh *et al.*	69.6% (50.5 – 85.0%)	4542	0.999
Chua *et al.*	90.9% (84.4 – 95.6%)	9093	0.999
Yusof *et al.*	68.8% (56.8 – 78.9%)	5228	1.000
$se_{LM}^{(Pk)}$			
Putaporntip *et al.*	15.1% (2.32 – 41.5%)	4935	1.000
Han *et al.*	51.0% (3.02 – 97.5%)	5736	0.999
Baum *et al.*	49.8% (2.64 – 97.7%)	5575	1.000
Cooper *et al.*	0.074% (0.0022 – 0.44%)	5268	0.999
Zhou *et al.*	20.8% (1.18. – 70.6%)	4949	0.999

Barber *et al.*	75.1% (58.4 – 86.3%)	2669	1.001
Barber *et al.*	33.5% (20.5 – 47.3%)	2528	1.001
Goh *et al.*	58.8% (40.3 – 77.0%)	3811	1.000
Chua *et al.*	76.5% (61.2 – 88.4%)	5115	0.999
Yusof *et al.*	68.6% (57.3 – 78.8%)	3992	1.000
$se_{LM}^{(Pm)}$			
Putaporntip *et al.*	10.4% (2.63 – 26.2%)	5095	1.000
Han *et al.*	71.1% (14.5 – 98.8%)	5801	0.999
Baum *et al.*	70.3% (15.7 – 98.5%)	5062	1.000
Cooper *et al.*	0.073% (0.0026 – 0.42%)	5128	1.000
Zhou *et al.*	6.60% (0.273 – 31.1%)	5145	0.999
Barber *et al.*	60.6% (5.52 – 98.3%)	5562	1.000
Barber *et al.*	59.6% (12.4 – 97.8%)	4893	1.000
Goh *et al.*	70.5% (17.8 – 98.6%)	4596	1.000
Chua *et al.*	78.8% (28.4 – 99.2%)	4948	1.000
Yusof *et al.*	25.1% (0.94 – 81.2%)	4125	1.000
$se_{LM}^{(Po)}$			
Putaporntip *et al.*	0.52% (0.021 – 3.14%)	4989	1.000
Han *et al.*	0.53% (0.020 – 3.39%)	4825	0.999
Baum *et al.*	0.52% (0.020 – 3.36%)	4897	0.999
Cooper *et al.*	0.50% (0.020 – 2.90%)	5376	1.000
Zhou *et al.*	0.52% (0.020 – 3.17%)	5214	1.000
Barber *et al.*	0.52% (0.021 – 3.33%)	4473	0.999
Barber *et al.*	0.52% (0.021 – 3.23%)	5107	0.999
Goh *et al.*	0.52% (0.021 – 3.19%)	4820	1.000
Chua *et al.*	0.55% (0.022 – 3.57%)	4822	0.999
Yusof *et al.*	0.51% (0.020 – 3.36%)	5127	0.999

Table S4. Posterior estimates and convergence statistics of study-level specifities when *P. knowlesi* and *P. malariae* LM samples are grouped.

Parameter	Posterior Estimate (95% CI)	Effective Sample Size	R-Hat
$sp_{LM}^{(Pf)}$			
Putaporntip *et al.*	95.6% (94.3 – 96.7%)	4825	0.999
Han *et al.*	97.3% (92.0 – 99.3%)	4754	1.000
Baum *et al.*	99.5% (98.6 – 99.9%)	4991	1.000
Cooper *et al.*	99.9% (99.9 – 100%)	2502	1.001
Zhou *et al.*	97.7% (95.9 – 98.8%)	7220	1.000
Barber *et al.*	93.8% (91.4 – 95.6%)	5605	1.000
Barber *et al.*	92.5% (88.6 – 95.3%)	5076	1.000
Goh *et al.*	96.0% (93.0 – 98.0%)	6313	1.000
Chua *et al.*	98.0% (95.0 – 99.3%)	5371	1.000
Yusof *et al.*	99.0% (98.1 – 99.5%)	7604	1.000
$sp_{LM}^{(Pv)}$			
Putaporntip *et al.*	96.5% (95.0 – 97.7%)	6215	0.999
Han *et al.*	98.1% (94.2 – 99.5%)	5321	1.000
Baum *et al.*	99.6% (98.7 – 99.9%)	4422	1.000
Cooper *et al.*	100% (99.9 – 100%)	2504	1.001
Zhou *et al.*	95.4% (92.5 – 97.4%)	7498	1.000
Barber *et al.*	96.8% (95.0 – 98.1%)	7300	0.999
Barber *et al.*	96.3% (94.0 – 97.9%)	4218	1.000
Goh *et al.*	97.9% (95.8 – 99.1%)	5298	0.999
Chua *et al.*	98.7% (96.3 – 99.6%)	5564	0.999
Yusof *et al.*	98.1% (96.8 – 98.9%)	6462	1.000
$sp_{LM}^{(Pk)}$			
Putaporntip *et al.*	100% (99.8 – 100%)	6011	0.999
Han *et al.*	99.2% (96.0 – 100%)	5370	1.000
Baum *et al.*	99.8% (98.8 – 100%)	6624	0.999
Cooper *et al.*	90.7% (86.4 – 93.9%)	2462	1.001
Zhou *et al.*	99.9% (99.3 – 100%)	5285	1.000
Barber *et al.*	77.7% (53.5 – 98.6%)	1854	1.001
Barber *et al.*	94.8% (87.6 – 99.7%)	2339	1.001
Goh *et al.*	97.3% (93.1 – 99.9%)	2830	1.000

Chua *et al.*	98.9% (96.2 – 99.9%)	4304	1.001
Yusof *et al.*	98.0% (95.0 – 99.9%)	2526	1.000
$sp_{LM}^{(Pm)}$			
Putaporntip *et al.*	100% (99.8 – 100%)	5306	1.000
Han *et al.*	99.2% (96.0 – 100%)	6341	1.000
Baum *et al.*	99.8% (98.8 – 100%)	5702	1.000
Cooper *et al.*	97.1% (95.6 – 98.2%)	2291	1.001
Zhou *et al.*	99.9% (99.4 – 100%)	5266	1.000
Barber *et al.*	73.7% (52.6 – 98.4%)	1857	1.001
Barber *et al.*	94.9% (87.6 – 99.7%)	2697	1.001
Goh *et al.*	97.3% (93.0 – 99.9%)	2866	1.001
Chua *et al.*	98.9% (96.3 – 99.9%)	3584	1.000
Yusof *et al.*	98.0% (95.1 – 99.9%)	2722	1.000
$sp_{LM}^{(Po)}$			
Putaporntip *et al.*	100% (100 – 100%)	4572	1.000
Han *et al.*	100% (100 – 100%)	4106	1.000
Baum *et al.*	100% (100 – 100%)	4334	1.000
Cooper *et al.*	100% (100 – 100%)	5774	1.000
Zhou *et al.*	100% (100 – 100%)	4122	1.000
Barber *et al.*	100% (100 – 100%)	4371	1.000
Barber *et al.*	100% (100 – 100%)	4249	1.001
Goh *et al.*	100% (100 – 100%)	3637	1.001
Chua *et al.*	100% (100 – 100%)	3962	1.001
Yusof *et al.*	100% (100 – 100%)	4123	1.000

Table S5. Posterior estimates and convergence statistics of group-level effects when *P. knowlesi* and *P. malariae* LM samples are not grouped.

Parameter	Posterior Estimate (95% CI)	Effective Sample Size	R-Hat
$\boldsymbol{\mu_{se}}$			
P. falciparum	0.191 (-0.562 – 0.978)	791	1.004
P. vivax	0.104 (-0.674 – 0.870)	1019	1.002
P. knowlesi	-3.27 (-4.47 - -2.36)	3266	1.000
P. malariae	-1.56 (-2.48 - -0.683)	4927	0.999
P. ovale	-5.61 (-9.00 - -3.82)	3798	1.000
$\boldsymbol{\sigma_{se}}$			
P. falciparum	1.17 (0.957 – 1.41)	3090	1.000
P. vivax	1.14 (0.930 – 1.41)	3035	0.999
P. knowlesi	0.571 (0.218 – 0.929)	3479	1.001
P. malariae	0.222 (0.0124 – 0.701)	2790	1.000
P. ovale	0.179 (0.00812 – 0.573)	5560	1.001
$\boldsymbol{\mu_{sp}}$			
P. falciparum	4.27 (3.56 – 4.99)	1096	1.003
P. vivax	4.52 (3.79 – 5.23)	928	1.005
P. knowlesi	5.88 (4.93 – 7.05)	2551	1.001
P. malariae	3.73 (2.99 – 4.56)	1189	1.002
P. ovale	12.4 (10.5 – 15.7)	2505	1.001
$\boldsymbol{\sigma_{sp}}$			
P. falciparum	1.03 (0.818 – 1.30)	3352	1.000
P. vivax	1.02 (0.811 – 1.28)	2736	1.001
P. knowlesi	0.933 (0.682 – 1.24)	4687	1.000
P. malariae	1.14 (0.914 – 1.42)	3886	1.000
P. ovale	0.176 (0.00826 – 0.596)	5598	1.001

Table S6. Posterior estimates and convergence statistics of study-level sensitivities when *P. knowlesi* and *P. malariae* LM samples are not grouped.

Parameter	Posterior Estimate (95% CI)	Effective Sample Size	R-Hat
$se_{LM}^{(Pf)}$			
Putaporntip *et al.*	83.5% (80.8 – 86.0%)	3948	1.000
Han *et al.*	89.5% (78.5 – 96.3%)	8035	1.000
Baum *et al.*	38.8% (23.0 – 55.9%)	10153	0.999
Zhou *et al.*	91.0% (86.4 – 94.6%)	4984	1.000
Goh *et al.*	49.0% (27.8 – 69.9%)	3331	1.000
Chua *et al.*	92.1% (85.9 – 96.3%)	7511	0.999
Yusof *et al.*	16.9% (7.51 – 35.2%)	2537	1.001
Cooper *et al.*	1.43% (0.799 – 2.42%)	5009	1.000
Barber *et al.*	50.8% (34.7 – 67.5%)	8310	0.999
Barber *et al.*	43.2% (27.8 – 60.1%)	4056	1.000
$se_{LM}^{(Pv)}$			
Putaporntip *et al.*	83.5% (81.3 – 85.6%)	4059	1.000
Han *et al.*	78.8% (59.0 – 91.7%)	9793	0.999
Baum *et al.*	57.3% (39.3 – 74.2%)	9129	1.000
Zhou *et al.*	90.5% (86.7 – 93.5%)	4008	1.000
Goh *et al.*	52.0% (29.0 – 75.6%)	3506	0.999
Chua *et al.*	90.7% (84.0 – 95.5%)	7018	1.000
Yusof *et al.*	15.2% (7.09 – 29.5%)	2475	1.001
Cooper *et al.*	1.44% (0.774 – 2.50%)	5361	0.999
Barber *et al.*	49.4% (38.1 – 61.0%)	5078	0.999
Barber *et al.*	29.2% (17.5 – 44.7%)	4186	1.000
$se_{LM}^{(Pk)}$			
Putaporntip *et al.*	3.36% (0.591 – 11.0%)	4603	0.999
Han *et al.*	3.70% (0.627 – 13.7%)	4265	1.000
Baum *et al.*	3.76% (0.655 – 13.9%)	4672	0.999
Zhou *et al.*	3.63% (0.626 – 12.7%)	4985	0.999
Goh *et al.*	1.97% (0.391 – 5.99%)	4314	1.000
Chua *et al.*	2.70% (0.539 – 7.95%)	4777	1.001
Yusof *et al.*	10.5% (5.00 – 19.4%)	2489	1.000
Cooper *et al.*	0.0449% (0.00159 – 0.297%)	5699	1.000
Barber *et al.*	68.6% (52.5 – 81.5%)	7299	0.999
Barber *et al.*	27.8% (16.2 – 41.7%)	4196	0.999
$se_{LM}^{(Pm)}$			
Putaporntip *et al.*	15.5% (6.48 – 29.3%)	7305	0.999
Han *et al.*	18.1% (7.28 – 43.9%)	4240	1.000
Baum *et al.*	18.3% (7.28 – 44.4%)	3820	1.000
Zhou *et al.*	15.6% (6.08 – 31.2%)	6673	0.999
Goh *et al.*	18.5% (7.54 – 43.2%)	4262	1.000
Chua *et al.*	19.1% (7.69 – 49.6%)	3434	1.000
Yusof *et al.*	16.2% (5.96 – 33.3%)	5325	1.000
Cooper *et al.*	0.0477% (0.00182 – 0.271%)	6007	1.000
Barber *et al.*	65.6% (12.4 – 98.3%)	8232	0.999
Barber *et al.*	58.1% (14.6 – 97.3%)	6232	1.000
$se_{LM}^{(Po)}$			
Putaporntip *et al.*	0.358% (0.0126 – 2.33%)	6071	0.999
Han *et al.*	0.357% (0.0122 – 2.33%)	6680	0.999
Baum *et al.*	0.353% (0.0123 – 2.32%)	6020	1.000
Zhou *et al.*	0.354% (0.0117 – 2.73%)	5687	0.999
Goh *et al.*	0.360% (0.0121 – 2.31%)	5873	0.999
Chua *et al.*	0.381% (0.0124 – 2.55%)	5557	0.999
Yusof *et al.*	0.354% (0.0121 – 2.26%)	6046	0.999
Cooper *et al.*	0.345% (0.0123 – 1.97%)	6773	1.000
Barber *et al.*	0.360% (0.0118 – 2.30%)	5397	1.000
Barber *et al.*	0.360% (0.0121 – 2.25%)	5912	1.000

Table S7. Posterior estimates and convergence statistics of study-level specificities when *P. knowlesi* and *P. malariae* LM samples are not grouped.

Parameter	Posterior Estimate (95% CI)	Effective Sample Size	R-Hat
$se_{LM}^{(Pf)}$			
Putaporntip *et al.*	95.6% (94.3 – 96.8%)	4094	1.000
Han *et al.*	97.6% (92.3 – 99.5%)	7006	1.000
Baum *et al.*	99.6% (98.7 – 99.9%)	5124	1.000
Zhou *et al.*	97.7% (96.1 – 98.8%)	7103	0.999
Goh *et al.*	96.8% (94.1 – 98.4%)	5146	0.999
Chua *et al.*	98.1% (95.3 – 99.4%)	8000	1.000
Yusof *et al.*	99.7% (99.3 – 99.9%)	3643	1.000
Cooper *et al.*	100% (99.9 – 100%)	4868	0.999
Barber *et al.*	93.9% (91.7 – 95.7%)	4379	1.000
Barber *et al.*	92.9% (89.1 – 95.7%)	4107	1.001
$se_{LM}^{(Pv)}$			
Putaporntip *et al.*	96.6% (95.1 – 97.7%)	4720	1.000
Han *et al.*	98.2% (94.4 – 99.6%)	7172	1.000
Baum *et al.*	99.7% (98.8 – 99.9%)	5059	1.000
Zhou *et al.*	95.5% (92.7 – 97.5%)	5898	0.999
Goh *et al.*	98.4% (96.6 – 99.4%)	6548	0.999
Chua *et al.*	98.8% (96.5 – 99.7%)	6531	0.999
Yusof *et al.*	99.4% (98.8 – 99.8%)	2780	1.000
Cooper *et al.*	100% (100 – 100%)	5132	0.999
Barber *et al.*	96.8% (95.1 – 98.1%)	6436	0.999
Barber *et al.*	96.6% (94.4 – 98.1%)	4535	1.000
$se_{LM}^{(Pk)}$			
Putaporntip *et al.*	99.9% (99.8 – 100%)	6702	1.000
Han *et al.*	99.8% (98.8 – 100%)	4284	1.000
Baum *et al.*	99.8% (99.3 – 100%)	4391	1.000
Zhou *et al.*	99.9% (99.5 – 100%)	5348	0.999
Goh *et al.*	99.8% (99.3 – 100%)	5087	1.000
Chua *et al.*	99.8% (99.1 – 100%)	5235	0.999
Yusof *et al.*	99.5% (98.8 – 99.8%)	3543	1.000
Cooper *et al.*	93.8% (90.1 – 96.4%)	4947	1.000
Barber *et al.*	99.7% (97.6 – 100%)	2717	1.001
Barber *et al.*	99.7% (96.7 – 100%)	979	1.005
$se_{LM}^{(Pm)}$			
Putaporntip *et al.*	99.8% (99.6 – 100%)	8692	1.000
Han *et al.*	99.2% (96.9 – 99.9%)	5342	1.000
Baum *et al.*	99.6% (98.6 – 99.9%)	7725	0.999
Zhou *et al.*	99.7% (99.1 – 99.9%)	6089	1.000
Goh *et al.*	89.1% (84.1 – 93.5%)	3904	1.000
Chua *et al.*	87.0% (82.3 – 90.7%)	4028	1.001
Yusof *et al.*	97.2% (95.0 – 98.7%)	2569	1.000
Cooper *et al.*	98.1% (96.9 – 98.9%)	5475	1.000
Barber *et al.*	61.8% (50.6 – 72.1%)	4132	1.000
Barber *et al.*	91.9% (87.3 – 95.9%)	2692	1.002
$se_{LM}^{(Po)}$			
Putaporntip *et al.*	100% (100 – 100%)	5006	1.000
Han *et al.*	100% (100 – 100%)	4559	0.999
Baum *et al.*	100% (100 – 100%)	4827	0.999
Zhou *et al.*	100% (100 – 100%)	4828	0.999
Goh *et al.*	100% (100 – 100%)	4950	0.999
Chua *et al.*	100% (100 – 100%)	4752	0.999
Yusof *et al.*	100% (100 – 100%)	4704	0.999
Cooper *et al.*	100% (100 – 100%)	5644	0.999
Barber *et al.*	100% (100 – 100%)	4806	0.999
Barber *et al.*	100% (100 – 100%)	4433	0.999

Validation of Results

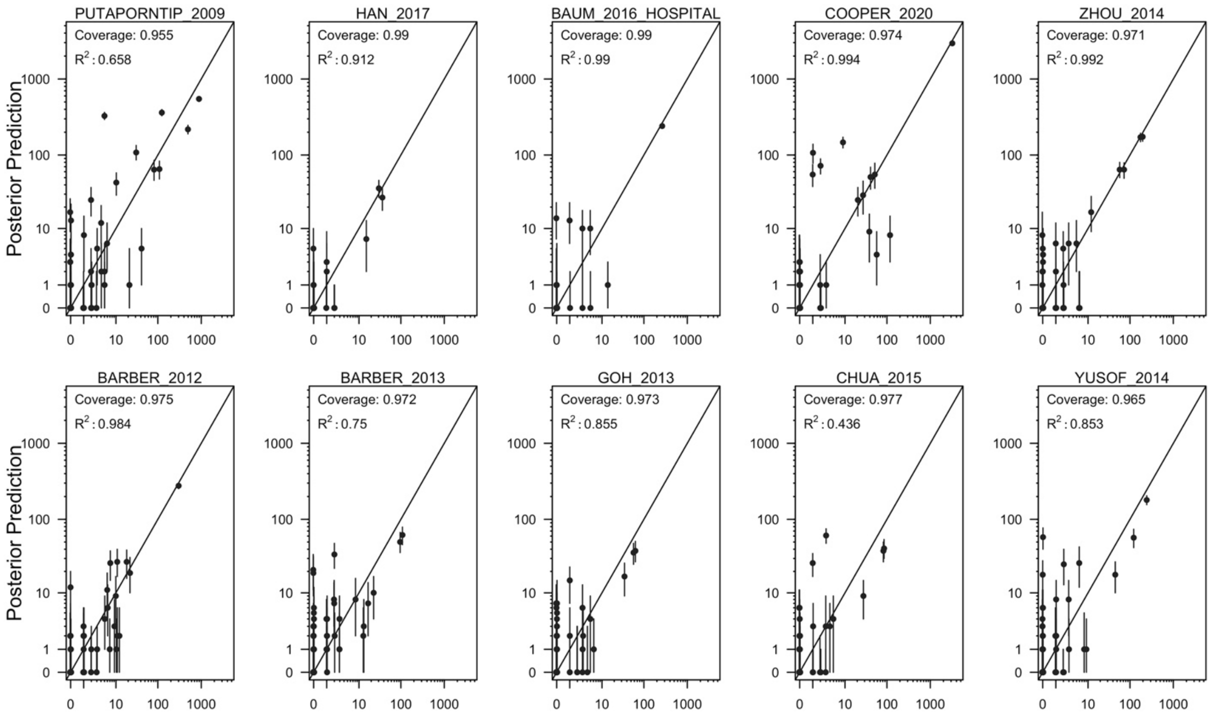

Figure S6. Posterior predictive check of fitted model in which P. knowlesi and P. malariae LM samples are grouped. *Comparison of the observed data and the posterior predictions are made for each of the studies included in model fitting. On the horizontal axis, each observation is a data point from a study, representing the number of samples that were observed with a given set of LM and PCR diagnostic outcomes across all Plasmodium spp. On the vertical axis, the point is the median posterior prediction, and the segment is the 95% prediction interval. The diagonal line in each plot is the one-to-one line, and the coverage probabilities and* R^2 *are reported for each study. Both axes are on a base-10 logarithm scale.*

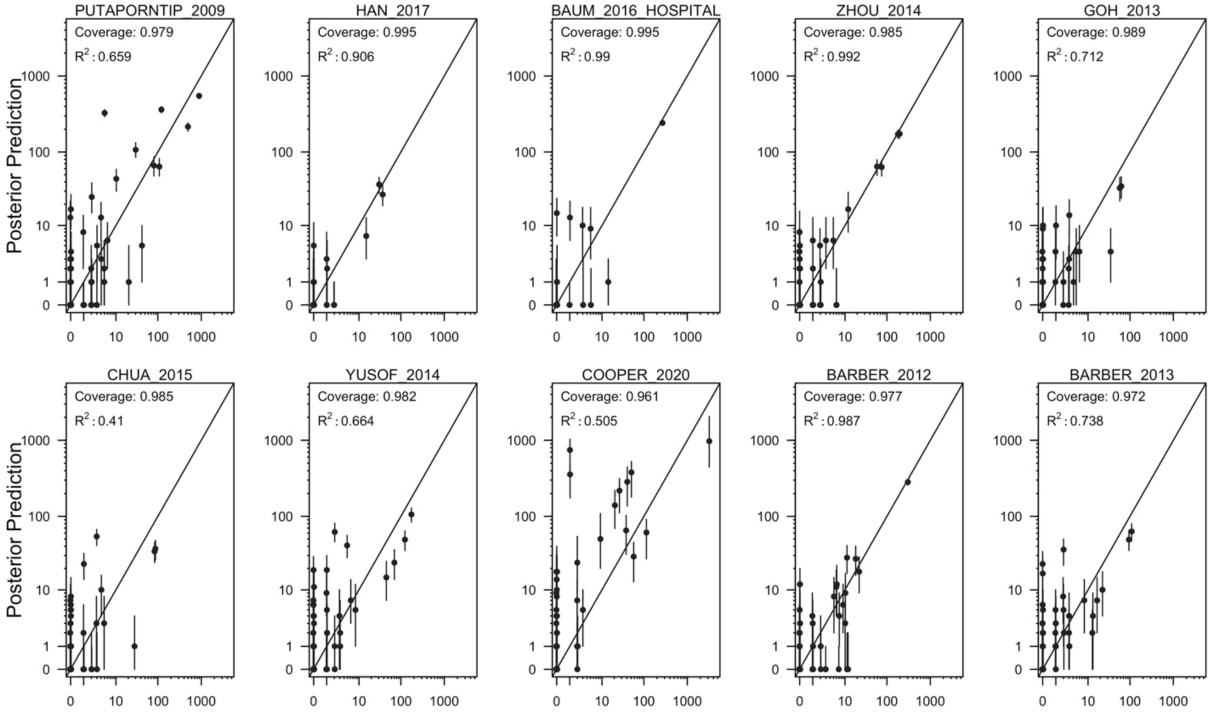

Figure S7. Posterior predictive check of fitted model in which P. knowlesi and P. malariae LM samples are not grouped. *Comparison of the observed data and the posterior predictions are made for each of the studies included in model fitting. On the horizontal axis, each observation is a data point from a study, representing the number of samples that were observed with a given set of LM and PCR diagnostic outcomes across all Plasmodium spp. On the vertical axis, the point is the median posterior prediction, and the segment is the 95% prediction interval. The diagonal line in each plot is the one-to-one line, and the coverage probabilities and* R^2 *are reported for each study. Both axes are on a base-10 logarithm scale.*

Sensitivity Analyses

Sensitivity to the Included Studies

To assess whether the inclusion of certain studies biased the results of our meta-analysis, we performed a sensitivity analysis for each study in which we re-fit the model while excluding that study. If the estimates of our hierarchical distribution means are robust to inclusion or exclusion of a given study, then that provides support that that study did not bias the parameter estimates that we obtained.

Our sensitivity analysis revealed that the hierarchical means are robust to the inclusion and exclusion of the studies (Fig. S8). For *P. falciparum*, *P. vivax*, and *P. malariae,* we observed variation in posterior median estimates of sensitivity and specificity for each study excluded. However, the uncertainty around these estimates was wide, and the 95% credible intervals overlapped with the estimates from the full analysis, indicating that our results were robust to the inclusion and exclusion of each study.

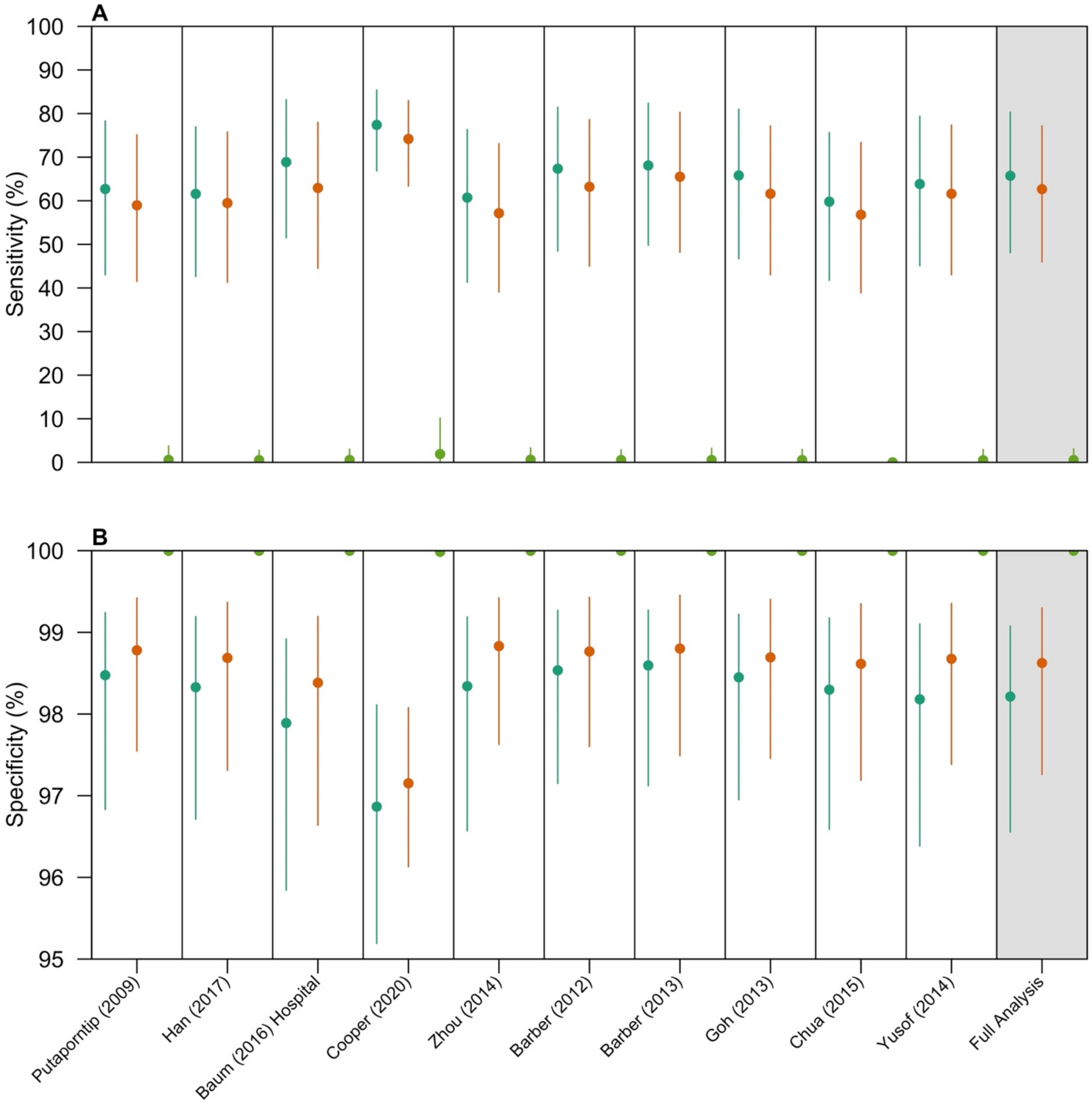

Figure S8. Sensitivity of hierarchical means to the included studies. *The hierarchical means for (A) sensitivity and (B) specificity are reported for the study excluded during model fitting. The colors denote the Plasmodium spp. considered, with teal representing P. falciparum, orange representing P. vivax, and green representing P. ovale. Each point is the posterior median estimate, and the vertical segment is the 95% credible interval.*

Sensitivity to the Assumption of PCR as the Gold Standard

In the primary analysis, we treated PCR as the gold standard and thus assumed that $se_{PCR}^{(k,l)} = 1$ and $sp_{PCR}^{(k,l)} = 1$ for all *Plasmodium* spp. k and studies l. To evaluate how robust our inferences

were to this assumption, we performed a sensitivity analysis in which we alternative assumed that $se_{PCR}^{(k,l)} = 0.95$ and $sp_{PCR}^{(k,l)} = 0.995$ for all *Plasmodium* spp. *k* and studies *l*. Accordingly, we computed the true parasite prevalence of *Plasmodium* spp. *k* in study *l* as

$$\theta_k^{(l)} = \frac{\hat{\theta}_k^{(l)} + sp_{PCR}^{(k,l)} - 1}{se_{PCR}^{(k,l)} + sp_{PCR}^{(k,l)} - 1}. \quad (S3)$$

In eq. (S3), $\hat{\theta}_k^{(l)}$ is the observed PCR prevalence when assuming PCR as the gold standard. The value of the specificity was chosen to ensure that $\theta_k^{(l)} > 0$ for all *Plasmodium* spp. *k* and studies *l*.

We found that our posterior estimates of the species-level sensitivities and specificities were mostly robust to the assumed values of PCR diagnostic performance (Fig. S9). Assuming an imperfect PCR method, our species-level estimate of sensitivity for *P. ovale* increased slightly, though the credible interval was wide and generally overlapped with the corresponding estimate obtained under the assumption of PCR as the gold standard. Our estimates of species-level specificity changed very little with the assumed values of PCR diagnostic performance.

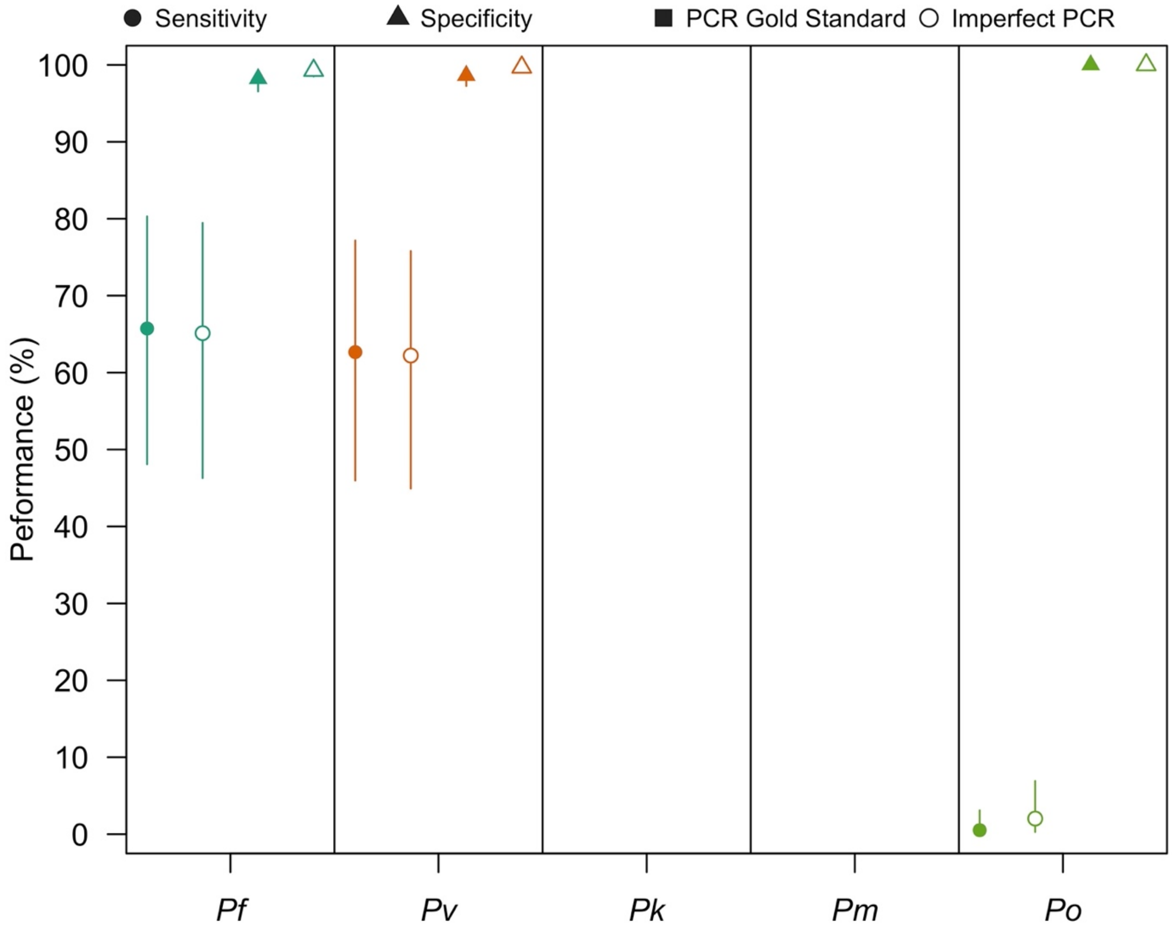

Figure S9. Sensitivity of LM diagnostic performance to the assumption of PCR as the gold standard. The posterior means for LM sensitivity (circles) and specificity (triangles) are shown when assuming PCR as the gold standard (filled points) and when assuming an imperfect PCR method (unfilled points) for each of the five *Plasmodium* spp. that causes human malaria. Each point is the posterior median estimate, and the vertical segment is the 95% credible interval.

Sensitivity to the Prior Distribution

In our inference framework, we placed a normal prior distribution with mean of zero and standard deviation of 0.25 on hierarchical standard deviation parameters. To evaluate whether our inferences were sensitive to the assumed standard deviation of this prior distribution, we re-fit our model, assuming values of prior distribution standard deviation from 0.125 to 1.0 in increments of 0.125.

In our sensitivity analysis, the hierarchical means for both sensitivity and specificity were robust to the assumed prior standard deviation (Fig. S10A & B). As the assumed standard deviation of the prior distribution increased, the posterior estimates for the hierarchical means increased only slightly. The width of the 95% credible interval increased with increasing prior standard deviation. That the hierarchical means for both sensitivity and specificity across the *Plasmodium* spp. were robust to the assumed prior distribution standard deviation further supports the conclusions reached in the primary analysis.

The estimates for the hierarchical standard deviations increased as the standard deviation on the prior distribution increased. This effect was more pronounced, given that the prior distribution was directly placed on these parameters. Although the estimate of the hierarchical standard deviation is sensitive to the prior assumption, the conclusions that we reached in the primary analysis do not depend strongly upon these parameters, so this should not affect the conclusions of our study.

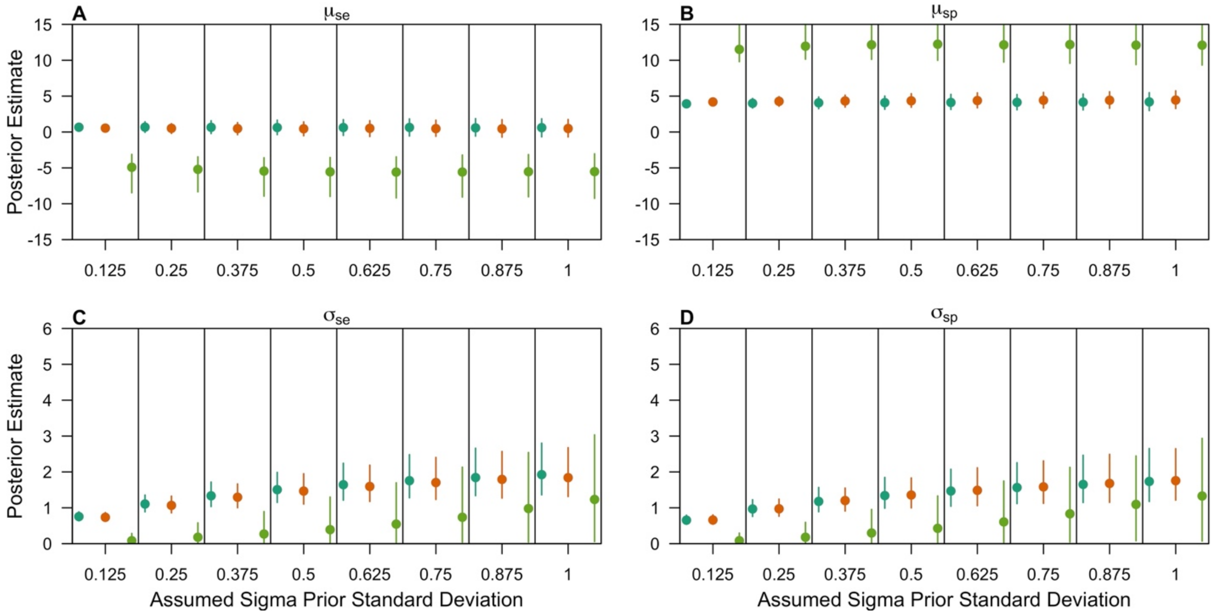

Figure S10. Sensitivity of hierarchical parameters to prior distribution assumption. *The sensitivities of the hierarchical mean for (A) sensitivity and (B) specificity and the hierarchical*

standard deviations of (C) sensitivity and (D) specificity are shown for each assumed prior standard deviation describing the hierarchical standard deviation parameters. In each plot, the point is the posterior median, and the vertical segment is the 95% credible interval. The color denotes the Plasmodium spp., with teal representing P. falciparum, orange representing P. vivax, and green representing P. ovale.*

Summary of the Study Samples

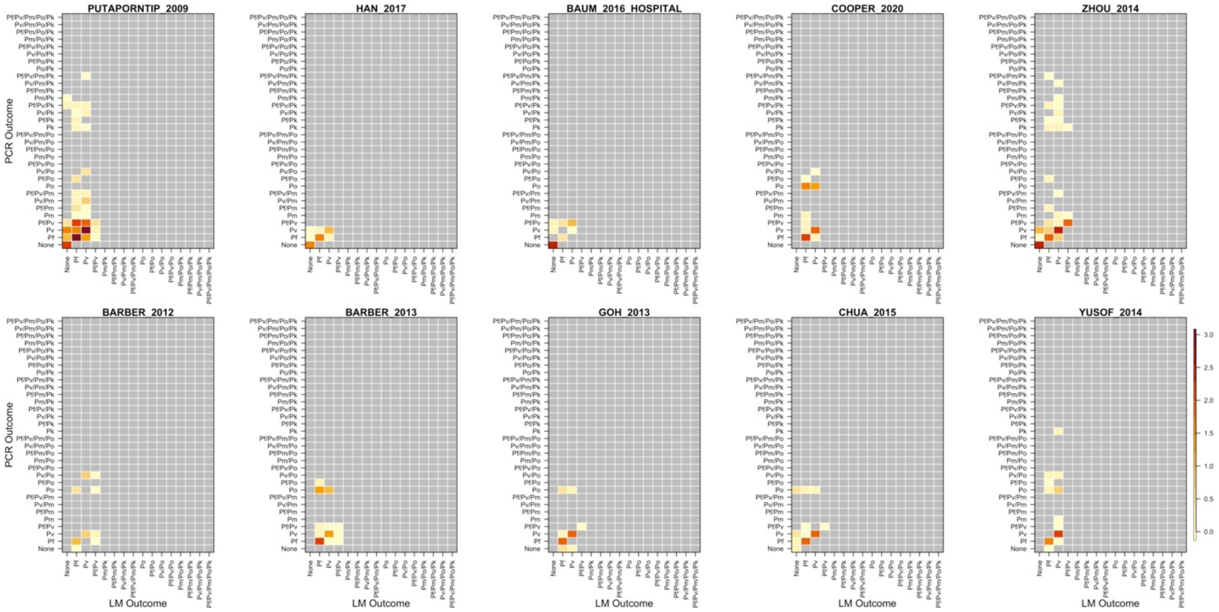

Figure S11. Heatmaps of study samples. *The base-10 logarithm of the number of samples by PCR and LM is reported for each study. The darker the color, the greater the number of observed samples, and gray indicates zero observed samples for that combination of LM and PCR. In this representation, P. knowlesi and P. malariae are grouped by LM.*

Testing the Temporal Effect on *Pk/Pm LM* Diagnosis

To test whether diagnosis of *P. knowlesi* and *P. malariae* infections as *"P. knowlesi / P. malariae*" has improved over time, we correlated the year in which data collection began for each study with the corresponding estimates of sensitivity and specificity (Fig. S12). We then tested the significance of each correlation. We found that the p-values were greater than 0.05 for all LM performance characteristics, indicating that the relationship with time was not statistically significant.

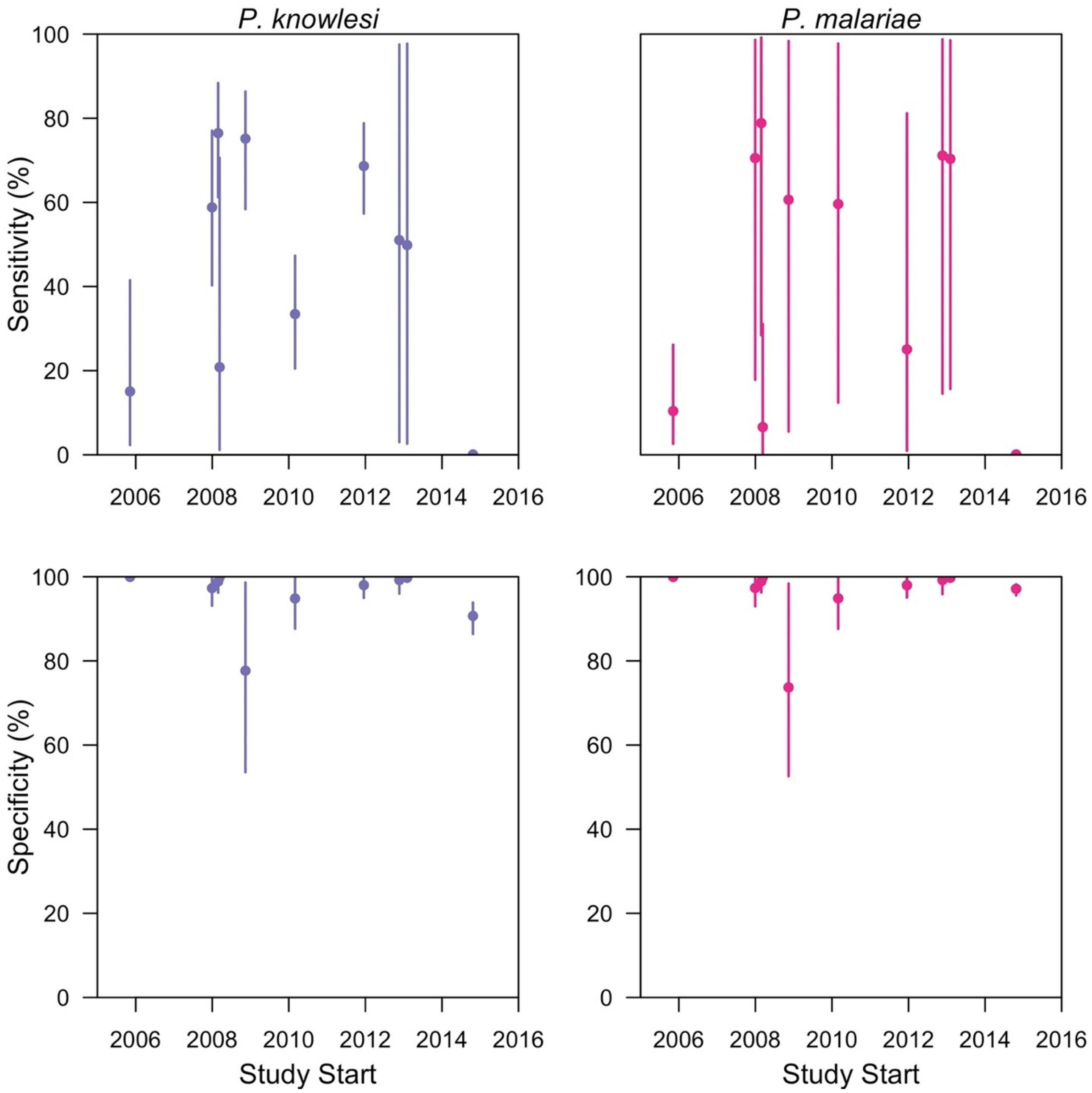

Figure S12. Temporal relationship of Pk and Pm LM sensitivity. *The sensitivity and specificity of LM for "Pk/Pm" given P. knowlesi (purple) and P. malariae (pink) are plotted against the starting year of data collection for each study. Points denote the median posterior estimate for each year, and bars denote the 95% credible interval.*

Sub-group Analysis by Study Design

Different studies included in our analysis employed different study designs to collect samples. Specifically, studies either included any febrile individual that presented in the health clinic, only febrile individuals that tested positive for *Plasmodium* spp. parasites by LM, or only febrile

individuals that tested positive for *Plasmodium* spp. parasites by both LM and PCR. We stratified the study-level estimates of LM sensitivity and specificity by study design. We found that, in general, there was no clear relationship between LM diagnostic performance and the study design. For *P. falciparum* and *P. vivax* LM sensitivity, it appears that study designs in which any febrile individual that presented to the health clinic was included in the sample yielded higher estimates of sensitivity with less uncertainty. This may reflect the fact that, by including individuals that ultimately tested negative for *Plasmodium* spp. by LM and/or PCR, we may obtain more certain estimates of sensitivity using the latent class model.

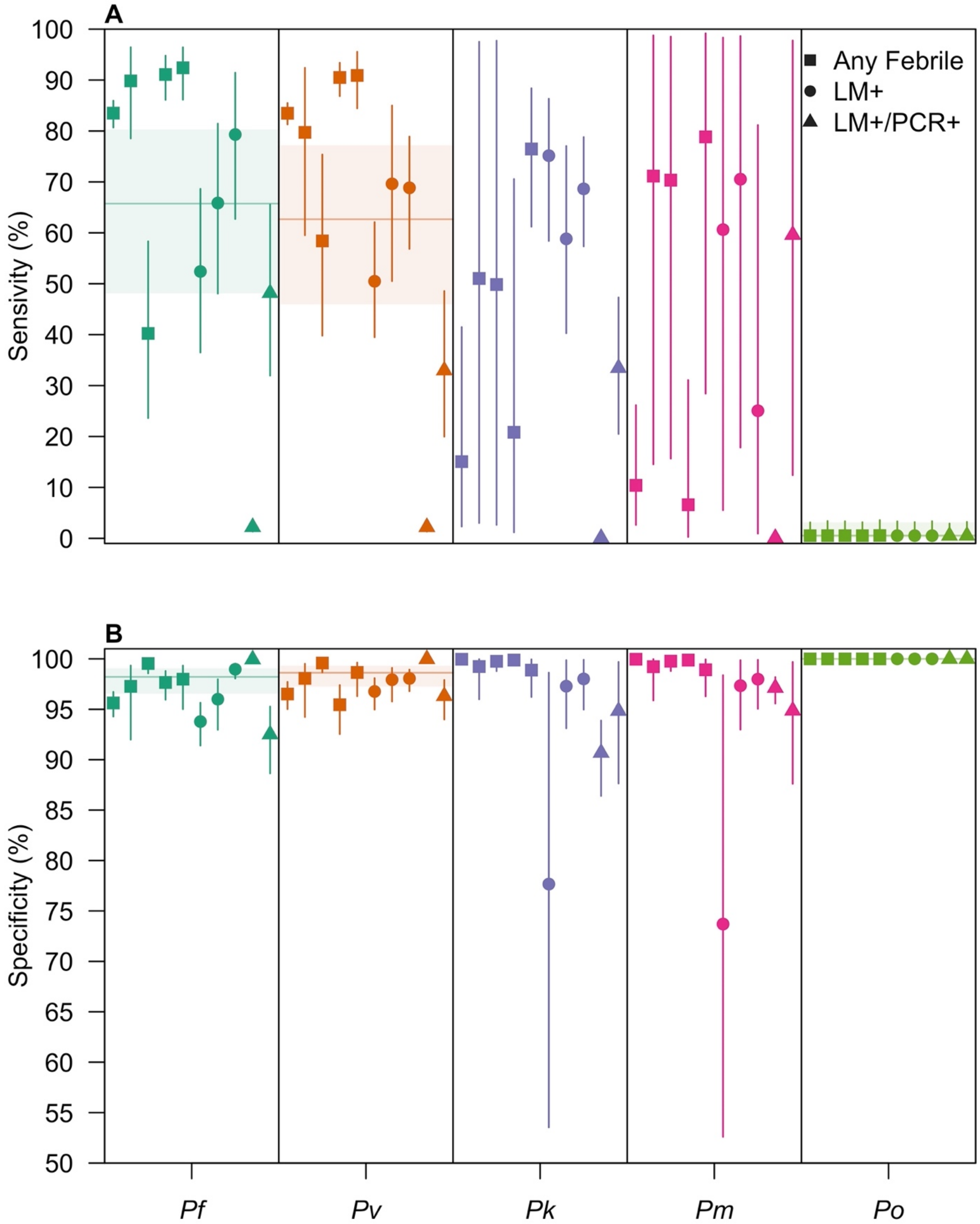

Figure S13. Study-level posterior estimates of LM diagnostic performance by study design. *The site-level posterior estimates of (A) LM sensitivity and (B) LM specificity are shown for P. falciparum (teal), P. vivax (orange), P. knowlesi (purple), P. malariae (pink), and P. ovale (green). Squares are study designs in which samples included any febrile individuals that presented to the health clinic, circles are study designs in which samples included only febrile*

individuals that tested positive by LM, and triangles are study designs in which samples included only febrile individuals that tested positive by both LM and PCR. The vertical segment is the 95% credible interval. The horizontal line is the posterior median of the group-level mean, and the horizontal shaded region is the corresponding 95% credible interval.
